# Psychometric properties of the ethical safety questionnaire in acute healthcare environments - a cross-sectional study

**DOI:** 10.1186/s12910-025-01279-1

**Published:** 2025-09-08

**Authors:** Adrienne Grech, Kati Naamanka, Janet Mattsson, Amir Pakpour, Suvi Kivelä, Maria Cassar, Katri Manninen, Kristaps Circenis, Agita Melbārde-Kelmere, Nina Korsström, Elena Marqués-Sule, Sara Cortés-Amador, David Hernández-Guillén, Tarja Poikkeus, Gunilla Björling

**Affiliations:** 1 Faculty of Health Sciences, University of Malta, Valetta, Malta; 2 Turku University of Applied Sciences, Turku, Finland; 3 Department of Health and Caring Sciences, Faculty of Health and Life Sciences, Linnaeus University, Växjö, Sweden; 4 Dept of Neurobiology, Care Sciences, and Society, Karolinska Institutet, Stockholm, Sweden; 5 School of Health and Welfare, Jönköping University, Jönköping, Sweden; 6 Department of Infectious Diseases, Karolinska University Hospital, Stockholm, Sweden; 7 Department of Learning, Informatics, Management, and Ethics, Karolinska Institutet, Stockholm, Sweden; 8 Faculty of Health and Sports Sciences, Department of Nursing and Midwifery, Riga Stradins University, Riga, Latvia; 9 Department of Physiotherapy, University of Valencia, Valencia, Spain; 10 Emergency Department and Prehospital Emergency Care, Kuopio University Hospital, Kuopio, Finland

**Keywords:** Ethical safety, Validation, Psychometric properties, Healthcare, Ethics

## Abstract

**Background:**

Ethical safety supports the development of ethical competencies and sound decision-making, central to good healthcare practice. The Ethical Safety Questionnaire (ESQ) evaluates ethical safety in healthcare environments, based on three subscales; *Ethical Autonomy*,* Ethical Respect*,* and Ethical Trust.* The objective of this study was to determine the psychometric properties of the ESQ in acute healthcare environments.

**Methods:**

The domain development of the ESQ was carried out in Finland in 2019. The ESQ was translated into English, Latvian and Swedish. To determine the validity of the ESQ, a cross-sectional design was used. Data was collected from nurses and physiotherapists (*n* = 275) in Sweden, Finland, Latvia and Malta, using purposive sampling. The validation (in all languages) consisted of an exploratory factor analysis, Rasch analysis and network analysis. This assessed the ESQ for internal consistency and construct validity. Ethical approval was granted from all participating countries in accordance with national law and the Declaration of Helsinki.

**Results:**

The internal consistency for the three subscales of the ESQ, *Ethical Autonomy*,* Ethical Respect*,* and Ethical Trust*, was satisfactory, with Cronbach’s α coefficients (> 0.7) and McDonald’s omega coefficients (> 0.7). All corrected item-total correlations among the items of the ESQ were high, exceeding 0.6. Thus, the ESQ exhibits acceptable levels of internal consistency and construct validity.

**Conclusions:**

The results suggest that the ESQ is a reliable tool for evaluating ethical safety in healthcare environments. However, the two general items in the ESQ can be omitted without loss of scale validity.

## Background

Ethical safety is defined as the independence of healthcare professionals to act according to their professional values. The ethical safety measure consists of four elements, including general ethical safety, ethical autonomy, ethical respect and ethical confidence. Ethical safety has been delineated as encompassing three critical dimensions: the patient, the professionals, and the environment. While the concept of ethical safety has been defined and studied to a limited extent [1]with a lack of research especially in physiotherapy, the importance of healthcare environments, contextual, and affective domains in supporting ethical decision-making and behaviour is well recognised [2].

Ethical safety in healthcare environments has been found to be supported by professionals’ ethical competence [3]ultimately improving both patient and professional outcomes [4]. This concept of safety from an ethical perspective revolves around providing care that upholds the patient’s rights, even when the patient is unable to self-advocate. Staff members who possess ethical competence demonstrate moral courage to tackle challenging issues, recognise the significance of ethical considerations, and seamlessly integrate ethical principles into their daily work contributing to such ethically safe environments. Creating an ethically safe environment is supported by organisation-wide discussions about ethics, regular updates to ethical practices and fostering open, continuous dialogue. Ethical safety in healthcare environments also requires respect and confidence in one’s ethical decisions by fellow healthcare professionals [1].

When healthcare professionals are aware of the ethically right decision but are unable to act upon it, they may experience moral distress. This distress contributes to ethically unsafe healthcare environments in view of the disconnect between ethical judgements and behavior [3]. Moral distress has been linked to burnout, diminished work satisfaction, decreased staff retention, decreased quality of patient care and impact on professional well-being [5–8].

For healthcare professionals to support ethically safe environments for both patients and staff, ethical competence is required. Ethical competence is understood in relation to ethical knowledge, ethical sensitivity, ethical reflection, ethical decision-making, and ethical action^3 9^. Being ethically competent supports high-quality patient care, ethical patient care and reduced moral distress among health professionals [5, 9]. Ethical competence can be achieved through adequate educational and organisational interventions [10].

Further to supporting the development of competence, educational interventions have also shown promise in increasing ethical safety for healthcare professionals and patients. The quality of patient care, as well as organisational outcomes, can be improved by education and clinical ethics interventions with health professionals [7, 11]. Promoting ethical safety by emphasising trust, building strong relationships, aligning around common goals, and actively facilitating participation, can significantly enhance ethical safety, at both institutional levels and for individual practitioners.

Thus, it is essential to be able to evaluate the level of ethical safety at organisations and among professionals, for the purposes of development and improvement of ethical care. The Ethical Safety Questionnaire (ESQ) specifically relating to healthcare environments is an instrument developed by Poikkeus [1]. The ESQ focuses on four components of safety, these include general ethical safety relating to the ability of nurses to provide good patient care, ethical autonomy which means that nurses are able to act independently according to their values. Ethical respect relating to nurses’ ethical decisions being respected by other professionals and interprofessional/intraprofessional ethical confidence. Ethical confidence relates to other professionals trusting and valuing nurses’ professional competence [4]. The ESQ was not previously psychometrically validated, and it is therefore necessary to evaluate the psychometric properties of this tool prior to further use. In the development of the ESQ, all statements were theoretically predefined based on the conceptual framework developed by Poikkeus (2019). The ESQ was afterwards validated for construct and face validity by an expert panel [1]. No analysis of the different factors within the scale and how they contribute to the overall ethical safety concept had been previously conducted.

### Objective

The objective was to validate and determine the psychometric properties of the ESQ in acute healthcare environments across four European countries.

## Methods

### Research design

The validation of the ESQ was carried out through a descriptive cross-sectional design study, where a purposive sampling was employed across the nursing and physiotherapy workforce in the four countries which participated in this study. Participants were recruited through intermediaries within one acute hospital in each partner country. These hospitals were selected based on their affiliation with the respective educational institutions. Participation in this research study was voluntary and anonymous. Data was collected from June 2023 to August 2023 via Webropol 3.0 in hospitals in Finland, Sweden, Latvia, and Malta.

### Participants and research context

#### Study population

The study population consisted of a total of 275 nurses and physiotherapists at four major hospitals in Finland, Sweden, Latvia, and Malta.

#### Inclusion and exclusion criteria

Nurses and physiotherapists in acute healthcare environments with permanent employment, both part-time and full-time, were eligible to participate in the present study. Healthcare professionals were included regardless of minimum work experience. Students and agency hired staff or temporary workers were excluded.

### Study contexts and recruitment procedure

Four major hospitals in Finland, Sweden, Latvia, and Malta were chosen for data collection. All hospitals were urban-based and nurses and physiotherapists working in acute care were invited to participate. Health professionals from non-acute settings were excluded. Purposive sampling was used to obtain the required sample across the four study settings. The hospitals involved in recruitment were all teaching hospitals affiliated with the educational entities conducting this research study. The respective management of the hospitals were informed about the study, and permissions were obtained to conduct the data collection. The researchers from each of the four countries, thereafter, introduced the study to the managers of the respective hospitals in the associated country and these managers acted as intermediaries by distributing the Webropol link to the questionnaire to all nurses/physiotherapists working at the hospital. Every participant received a link to the questionnaire with a cover letter informing participants about the aim and purpose of the study. The letter explained that participation entails the completion of a questionnaire (the ESQ) and it emphasised voluntary participation and assured anonymity in relation to participation. Completion of the questionnaire via Webropol implied a respondent’s consent to participate. A reminder to fill out the questionnaire was sent twice at two-week intervals.

### Sample size

Purposive sampling was used to obtain the required sample over 200 participants completed the questionnaires. This sample size complies with validation studies standards ranging from 100 to 400 [12].

### Data collection and instrument

#### Data collection tool

In this study, the ESQ was used [4]. Permission to translate and use the tool was granted by the developer, Tarja Poikkeus (also co-author of the present manuscript). The ESQ contains 11 items. Two items are general ethical safety statements and there are three subscales: ethical autonomy, ethical respect, and ethical confidence. The ESQ uses a 5-point Likert scale where 1 represents ‘strongly disagree’ up to 5 ‘strongly agree’. The three subscales all contain three items each. Higher scores on each ESQ subscale (Ethical Autonomy, Ethical Respect, and Ethical Trust) reflect stronger perceptions of ethical safety in the healthcare work environment.

#### Cross-cultural adaptation and face validity

The original ESQ in Finnish, was translated according to translation unit standards [13]. The forward translations (Finnish to English, Swedish, and Latvian) were completed by three translators who were fluent native speakers of the respective languages. This was followed by a reconciled translation based on the three translations; a third person, reviewed the translations and combined them into one reconciled ESQ. The reconciled ESQ-translated versions were back-translated into Finnish by two independent persons, for the respective language, who were fluent in English, Swedish and Latvian. The results of all steps (i.e., two forward translations, reconciliation, and two backward translations with comments) were proofread by an external proofreader who audited the final version of the ESQ in English, Swedish and Latvian.

### Pilot testing

The translated versions in Swedish, English, Latvian were ready for linguistic validation through pilot testing. The pilot tests of the translated versions were conducted in each country [13]. The questionnaire was completed by 10 participants in each country consisting of nurses and physiotherapists for face validation of the ESQ. Only minor changes were made after the piloting, such as changing terminologies to incorporate both physiotherapists and nurses within the specific healthcare environments.

### Psychometric evaluation

#### Statistical analysis

Items distributions are presented as frequency and percentage. Skewness and kurtosis statistics were computed to assess the distributional characteristics of the items. Values outside the range of −2 to + 2 for skewness and − 7 to + 7 for kurtosis were considered as potential deviations from normality [14].

The internal consistency of the ESQ was measured using Cronbach’s α and McDonald’s ω, with values higher than 0.7 indicating acceptable internal consistency. The item-total correlation (corrected for overlap) was further computed to measure the internal consistency with values higher than 0.4 indicating satisfactory consistency [15, 16].

The factor structure of the 9 items of the ESQ was assessed using exploratory factor analysis (EFA) with maximum likelihood extraction and varimax rotation. Sampling adequacy and the suitability of the correlation matrix were assessed using the Kaiser-Meyer-Olkin (KMO) test and Bartlett’s test of sphericity, respectively. A value higher than 0.6, along with a significant Bartlett’s test of sphericity, indicates the factorability of the data [17]. Significant factor loadings with a magnitude of > 0.40 were considered meaningful. The Scree plot and Kaiser rule were used to determine the number of factors to retain.

To investigate whether the items of the ESQ correspond with their underlying concept, a Rasch analysis using a partial credit model (PCM) was conducted [14]. Item fit was measured using indices including Outfit and infit mean square (MnSq) with values between 0.5 and 1.5 indicating good fit [18]. Local independence between the items was assessed by computing residual correlations between any two items of ESQ, with values higher than 0.2 indicating a violation of local independence. Differential item functioning (DIF) was computed to measure the measurement invariance of the ESQ items across gender and profession subgroups. A DIF contrast of 0.64 was set as a cut-off for a meaningful statistically significant DIF [19].

Finally, to evaluate the relationship between the ESQ items and general ethical safety, a network analysis was conducted. Network analysis is valuable for examining complex relationships and interactions between study variables and visualising these associations. In network analysis, each variable can be represented as a node, and the association between each node is referred to as an edge. The centrality of the nodes was determined by analyzing three metrics including degree centrality, betweenness centrality, and closeness centrality. The network analysis was computed using EBICglasso (Extended Bayesian Information Criterion Graphical Least Absolute Shrinkage Selection Operator) estimation, employing the non-parametric bootstrap procedure with 500 iterations to analyse the 95% confidence interval (CI) of edge weights [20]. All statistical analyses were performed using SPSS version 27, JASP version 0.18.3.0 and Winsteps version 4.3.0.

### Ethical considerations

The study follows the guidelines on research from the Declaration of Helsinki, regarding research on human subjects [18]. The respective University Ethics Committees approved the present study, including the Research Ethics Committee of Turku University of Applied Sciences (1/2023), Riga Stradins University Research Ethics Committee (2-PEK-4/507/2023) and the University of Malta, Faculty of Health Sciences Research Ethics Committee (FHS-2023-00077), and ethical guidelines according Swedish law (SFS: 2003:460) was followed, and accordingly approval to conduct the study was gained by the head of the clinics of the involved hospital. Additional permissions were addressed depending on specific country’s requirements. Furthermore, permissions were obtained from the executive directors of the respective study sites and the directors of the departments or head nurses. Informed consent was obtained from individual participants through completion of the questionnaire and participation was voluntary and anonymous; participants could withdraw their participation if they do not submit the survey.

## Results

### Sociodemographic characteristics of the study participants

As indicated in Table 1, the study involved 275 healthcare professionals with a mean age of 44.4 years. Most participants were from Latvia (34.2%) and the vast majority, 80 of the participants were nurses. The participants’ characteristics are displayed in Table 1.

**Table 1:** Participants’ characteristics (*N* = 275)

	Mean (± SD) or *n* (%)
Age (Year)	44.41 (± 12.79)
Gender	
Female	234 (85.1%)
Male	33 (12.0%)
Non-binary	2 (0.72%)
Do not want to disclose	6 (2.18%)
Country	
Sweden	57 (20.7%)
Malta	54 (19.6%)
Finland	70 (20.7%)
Latvia	94 (34.2%)
Profession	
Physiotherapist	54 (19.6%)
Nurse	221 (80.4%)
Academic qualification	
Diploma/Higher Diploma	50 (18.2%)
Bachelor’s Degree	125 (45.5%)
Master’s Degree/Postgraduate certificate/Diploma	96 (34.9%)
Doctoral Degree	2 (0.72%)
Years of work experience	19.92 (± 13.43)

### Psychometric evaluation

The distribution of responses to each ESQ item is presented in Table 2. The participants showed the highest agreement on items 1 and 2. Notably, no ceiling effects were observed, as none of the items received more than 50% of responses indicating “strongly agree.” There was no evidence of skewness or kurtosis in all ESQ items (Table 3), as indicated by values for skewness and kurtosis being between − 2 and + 2.

**Table 2:** Distribution of responses to the ethical safety instrument

Item #	Strongly disagree *N* (%)	Disagree *N* (%)	Neither agree nor disagree *N* (%)	Agree *N* (%)	Strongly agree *N* (%)
1. I am able to openly share my views on ethical problems *	1 (0.4%)	19 (6.9%)	54 (19.6%)	104 (37.8%)	95 (34.5%)
2. In my work, I am able to act in accordance with my own professional values*	5 (1.8%)	14 (5.1%)	43 (15.6%)	115 (41.8%)	95 (34.5%)
3. I am able to openly participate in discussions about what the common values and principles are*	5 (1.8%)	19 (6.9%)	47 (17.1%)	112 (40.7%)	91 (33.1%)
4. The ethical competence of the nurses/physiotherapists is valued in my organisation**	24 (8.7%)	41 (14.9%)	66 (24.0%)	77 (28.0%)	61 (22.2%)
5.Valuing nurses’/physiotherapists’ viewpoints on ethics**	27(9.8%)	44 (16.0%)	77(28.0%)	70 (25.5%)	51 (18.5%)
6. Nurses’/Physiotherapists’ participation in dealing with ethical problems is valued in my organisation**	28(10.2%)	47(17.1%)	87(31.6%)	57(20.7%)	53(19.3%)
7. Allied healthcare professionals trust my ability to make ethical decisions***	6(2.2%)	18(6.5%)	56(20.4%)	119(43.3%)	74(26.9%)
8. My managers trust me in my ability to make ethical decisions***	7 (2.5%)	21(7.6%)	43(15.6%)	102(37.1%)	97 (35.3%)
9. Medical professionals trust my ability to make ethical decisions***	11(4.0%)	16(5.8%)	52(18.9%)	111(40.4%)	81(29.5%)

^*^Ethical autonomy

^**^Ethical respect

^***^Ethical trust

**Fig. 1:**
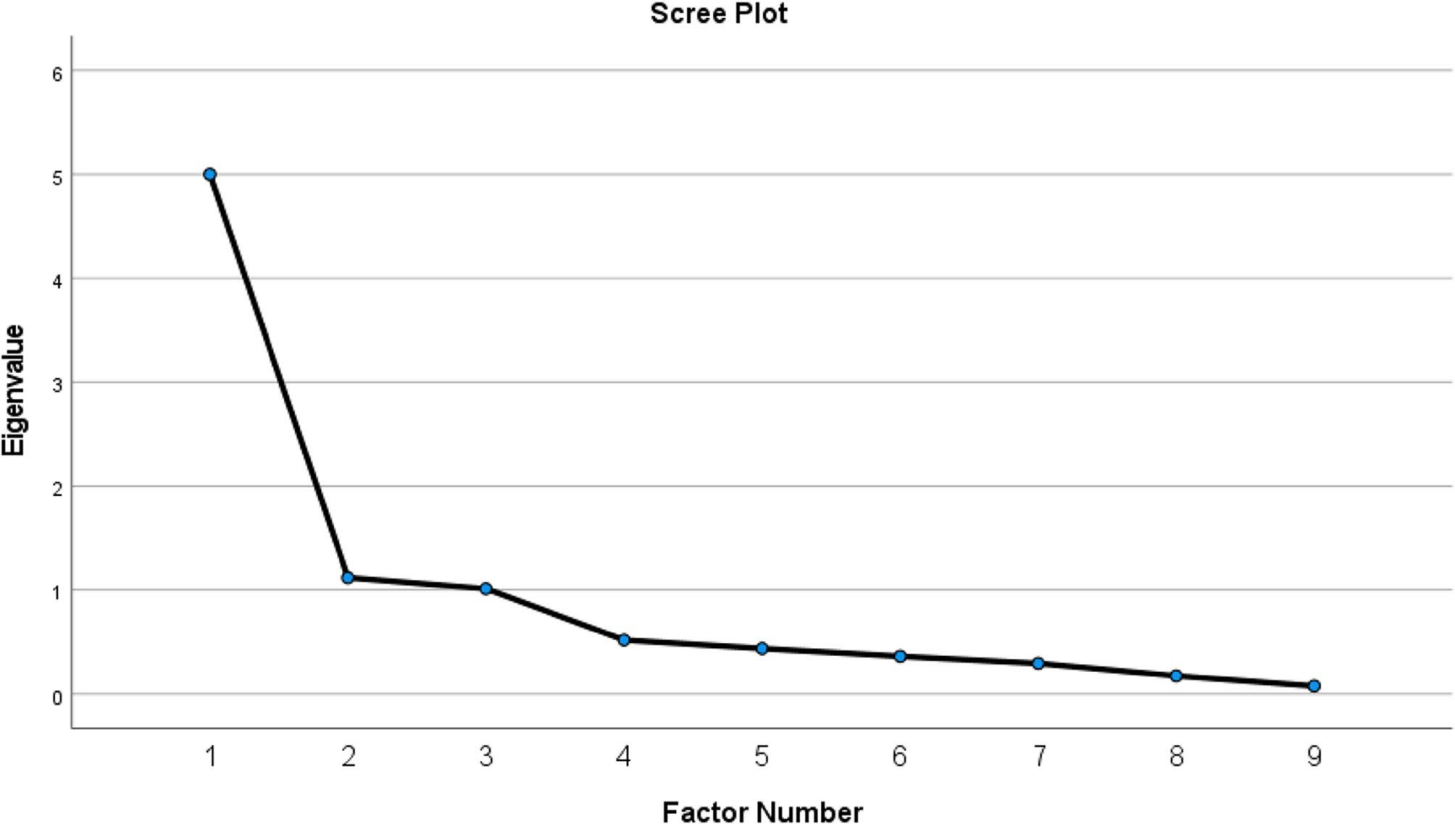
Scree plot with eigenvalues

The results of EFA are shown in Table 3. The factorability of the data was confirmed by interpreting the result of EFA, with KMO of 0.855, and Bartlett’s test p-values less than 0.001. Three factors were extracted, accounting for a total of 69.5% of the explained variance, respectively. Additionally, the screen plot indicated that the first component had an eigenvalue almost five times higher than the next two components. However, Factor 2 and 3 also exhibited eigenvalues visually higher than 1 (Fig. 1). Factor 1 (ethical autonomy) comprised three items: items 1, 2, and 3, while factor 2 (ethical respect) included items 4, 5, and 6. The final factor (ethical trust) consisted of items 7,8 and 9. All factor loadings were significant and higher than 0.40 (Table 3).

**Table 3:** Psychometric properties of the ethical safety instrument (ESQ) in item level

Item #	Analyses from classical test theory				Analyses from Rasch				
	Mean (standard deviation)	Skewness (kurtosis)	Factor loading^a^	Item-total correlation	Infit MnSq^e^	Outfit MnSq^e^	Difficulty	DIF contrast across gender^bc^	DIF contrast across profession^bd^
ESQ1	4.0 (0.93)	−0.64(−0.32)	0.644	0.633	0.80	0.76	0.13	0.72	0.24
ESQ2	4.03 (0.94)	−0.98(0.83)	0.645	0.625	1.07	1.0	−0.01	−0.31	−0.13
ESQ3	3.97 (0.97)	−0.87(0.37)	0.829	0.734	1.11	1.10	−0.12	−0.39	−0.11
ESQ4	3.41 (1.24)	−0.38(−0.83)	0.799	0.880	1.13	0.91	0.64	0.49	0.34
ESQ5	3.28 (1.23)	−0.25(−0.85)	0.906	0.937	0.61	0.59	0.14	0.49	−0.03
ESQ6	3.22 (1.24)	−0.13(−0.88)	0.804	0.879	1.13	1.04	−0.78	0.48	−0.29
ESQ7	3.87 (0.96)	−0.78(0.40)	0.684	0.647	0.97	0.97	0.09	−0.45	−0.28
ESQ8	3.97 (1.03)	−0.93(0.33)	0.638	0.640	1.13	1.05	−0.22	−0.05	0.29
ESQ9	3.87 (1.04)	−0.93(0.55)	0.771	0.693	0.90	0.82	0.13	−0.45	

a) Based on exploratory factor analysis

b) DIF contrast >0.5 indicates substantial DIF

c) DIF contrast across gender=Difficulty for males-Difficulty for females

d) DIF contrast across profession=Difficulty for nurses-Difficulty for physiotherapist.

e) *MnSq *Mean square error, *DIF* Differential item functioning

The internal consistency for the three subscales of the ESQ, namely Ethical Autonomy, Ethical Respect, and Ethical Trust, was satisfactory, with Cronbach’s α coefficients (> 0.7) and McDonald’s omega coefficients (> 0.7) indicating acceptable reliability (Table 4). All corrected item-total correlations among the items of the ESQ were high, exceeding 0.6.

**Table 4:** Internal consistency of the ethical safety instrument (ESQ)

Psychometric testing	Ethical autonomy	Ethical respect	Ethical trust	Suggested cutoff
*Internal consistency (Cronbach’s α)*	0.812	0.952	0.810	> 0.7
*McDonald’s omega*	0.821	0.953	0.812	> 0.7

The results of the Rasch analysis are presented in Table 3. All nine items of the ESQ demonstrated acceptable fit with the data, as evidenced by both Infit and Outfit MnSq falling between 0.5 and 1.5. Respondents identified item 4 (as the most difficult, while item 6 was deemed the easiest. No notable DIF was found across subgroups of participants based on gender and profession for all ESQ items. However, male participants had higher difficulty in item 1 compared to female participants.

**Fig. 2:**
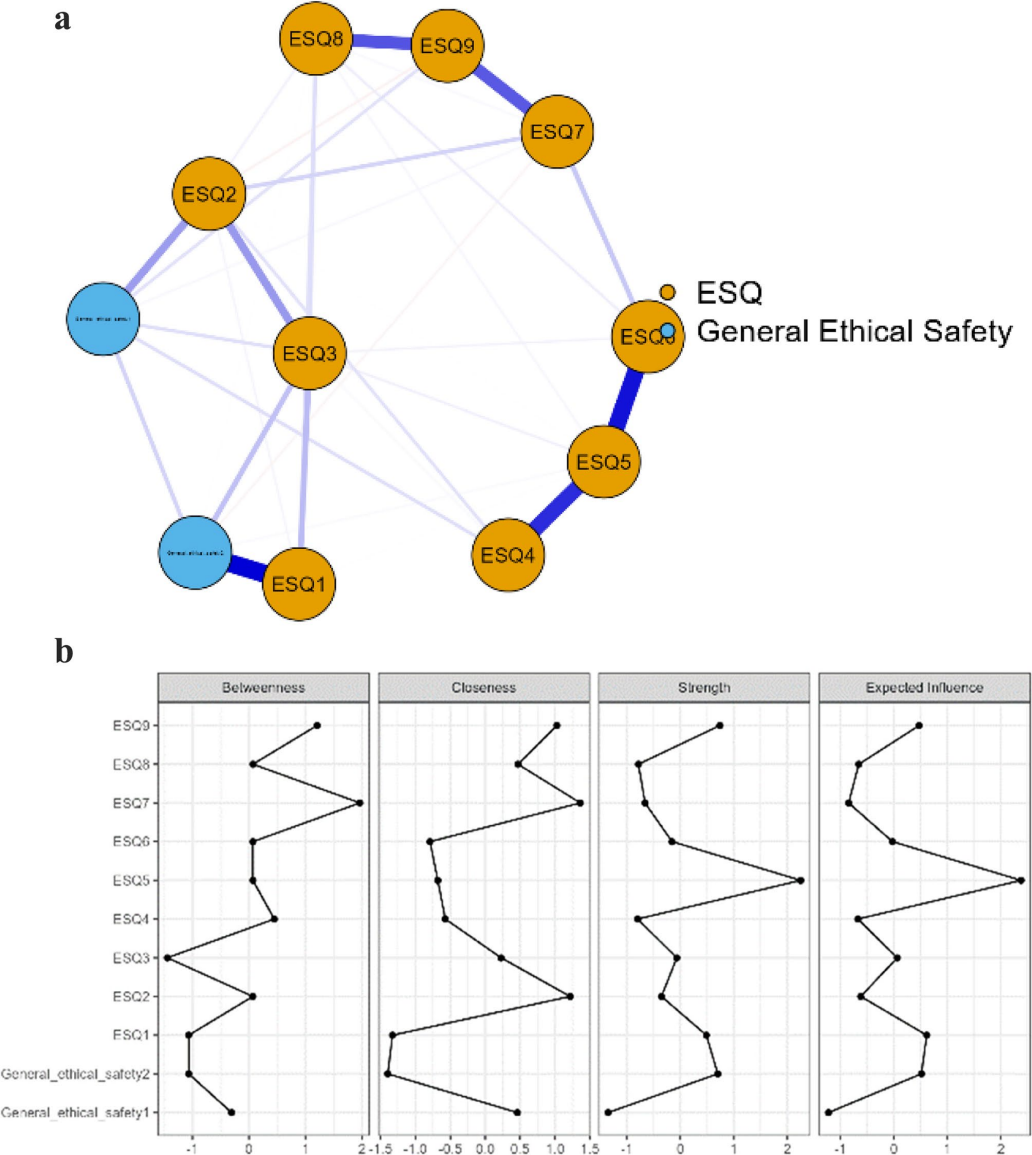
**a** Network plot of the ESQ items and general ethical safety. edges represent partial correlations between the nodes, where thicker edges represent stronger connections. Blue edges represent positive correlations, whereas red edges represent negative correlations. **b** The image below shows centrality measures for the ESQ items and general ethical safety questions

The relationship between overall ethical safety and ESQ items is visualized in Fig. 2 via network analysis. The network had 33 non-zero edges with a sparsity of 0.400. Items 5, 7, and 9 of the ESQ exhibited the highest values in centrality measures, indicating their significance within the network model. As presented in Fig. 2, the strongest connections were observed between item 1 ESQ1 and general ethical safety (*r* = 0.719), items 5 and 6 of ESQ (*r* = 0.670), as well as items 4 and 5 of ESQ (*r* = 0.599).

## Discussion

The aim of this study was to determine the psychometric properties of the ESQ in acute healthcare environments across four European countries. The ESQ, originally developed in Finnish, was translated to Swedish, English and Latvian for the purposes of this study. The original Finnish version of the ESQ had been tested for construct validity. This study entailed further analysis of the psychometric properties of the ESQ in a cross-cultural context. The overall results suggest that the ESQ offers a psychometrically sound, internally consistent and content-valid measure of ethical safety for nurses and physiotherapists in a cross-cultural context. KMO and Barthlett scores showed that an EFA was applicable.

The internal consistency for the three subscales of the ESQ, namely Ethical Autonomy, Ethical Respect, and Ethical Trust, was satisfactory, with Cronbach’s α coefficients (> 0.7) and McDonald’s omega coefficients (> 0.7) indicating internal consistency (Table 4). This indicates that the items within the ESQ are measuring the same dimensions they are intended to measure-ethical autonomy, ethical respect and ethical trust.

The EFA of the three subscales in the questionnaire showed that the data is unidimensional with all subscales contributing to an evaluation of ethical safety, corresponding closely with the predefined subscales. The internal consistency of the tool indicates that the subscales are drawing upon the same underlying concept of ethical safety, making the ESQ a reliable tool. Thus, ethical autonomy, ethical respect and ethical confidence are viewed as one approach to ethical safety within healthcare environments [4]. General ethical safety items were removed from the EFA as they were redundant and measured similar aspects of ethical safety, relating to the broader significance of ethical safety within organisations. This is also evidenced within the network analysis, in which all 9 items relate to the general ethical safety statements. The two general ethical safety items were removed because they were conceptually distinct from the rest of the scale. The remaining statements in the ESQ were grouped into three specific subscales, the two general items were broader in scopre and overalapped with the content of the three subscales. Namely, statement one was very vague referring to being able to provide ethically sound care, which is a general definition of ethical safety, whilst statement 2 was very similar to statement 3. This suggests that general ethical safety statements can be removed from the tool to provide an increasingly targeted measure of ethical safety, whilst still maintaining an appropriate measure for ethical safety across distinct dimensions.

An interpretation of the Rasch models can be described as a healthcare professional with lower scores on the ESQ, may indicate a lower perception or awareness of ethical safety in healthcare environments. As a result, this might suggest a limited understanding of ethical safety, poor ethical sensitivity in or lack of consideration towards ethical implications related to practice. This might indicate that participants who score at the lower end of the ESQ scale are less likely to contribute towards creating an ethically safe environment. This can be derived from James Rests four component model whereby moral reasoning is part of the psychological processes related to moral action [2]. A healthcare professional scoring at the higher levels on the ESQ can be described as having recognition of ethically safe environments and being more likely to contribute positively to such environments.

The network analysis revealed a dense network structure among the items for the ESQ, indicating interconnectedness among the various items of ethical safety. Valuing nurses’ viewpoints in ethics (item 5), trust in the ability to make ethical decisions by other professionals (item 7), trust from doctors in ethical decision-making (item 9), demonstrated the highest centrality. This suggests that these aspects have a significant impact in shaping the perceptions of ethical safety within organisations. The organisational aspect of healthcare practice has a significant impact on nurses’ experiences of ethics in practice. Healthcare organisations that are challenged with busy environments and organisational structure might cause nurses to compromise on their values and inhibit decision-making abilities [21]. Furthermore, professional autonomy showed that good nurse-physician relationships resulted in improved nurse autonomy [22, 23]. Lack of respect and collaboration with physicians and having hierarchical physician centered organisations were linked to inhibited decision-making and autonomy by nurses [24–26].

The network analysis further supports the importance of interdisciplinary ethical decision-making, with value being placed on nurses’ and physiotherapists’ perspectives about ethical issues. This can enhance the ability of healthcare professionals to uphold ethical standards and navigate ethical issues effectively when trusted by other professionals and having organisations that value the perspectives of health professionals. However, through the ESQ this finding can also indicate the significant hierarchical structure within healthcare environments, may influence ethical safety. Thus, further research is indicated to explore how power dynamics and organizational hierarchies specifically influence ethical safe environments. Existing literature suggests that hierarchical structures in healthcare organizations can impact ethical decision-making by influencing professional autonomy, interprofessional relationships, and the ability to voice ethical concerns [21, 22]. Validation results suggests that future research could further investigate how varying degrees of hierarchy either facilitate or hinder ethical safety, potentially drawing from qualitative studies that examine the lived experiences of healthcare professionals. Incorporating findings from previous research on organisational ethics and decision-making frameworks may provide additional insight into how power dynamics shape ethical environments. Strengthening this discussion would offer a more comprehensive understanding of the contextual factors influencing ethical safety within healthcare environments.

Willingness to share views on ethical issues (item 1) showed the strongest correlation to general ethical safety statements, compared to other items in the ESQ. This item highlights aspects of communication and ethical deliberation. The willingness of different healthcare professions to share their own opinions and perspectives on ethical issues contributes to an ethically safe environment. This supports the proposition of having safe spaces for healthcare professionals to discuss ethical issues [27, 28]. However, moral spaces and discussions require a genuine recognition of the healthcare professional’s ethical viewpoints as followed by item 5 (valuing nurses’ and physiotherapists’ viewpoints on ethics) and item 6 (valuing nurses’ and physiotherapists’ participation in ethics).

Item 4 (valuing nurses’ and physiotherapists’ ethical competence) had the highest difficulty score according to the MnSq. This suggests that participants who highly relate to concepts within ethical safety are more likely to answer this statement positively. Item 6 (Valuing nurses’ and physiotherapists’ participation in ethics) had the lowest difficulty score, suggesting that even participants who are not sensitive to ethical safety are likely to endorse this question as strongly agree. There is a wide consensus in the literature about nurses’ participation in ethics [1, 2]. Thus, this statement is unlikely to result in any disagreement, even by individuals who have limited knowledge of ethics in practice [4, 21].

Item 1–3,7–9 (covering ethical safety and ethical trust) are the items that individuals with a lower ethical safety score are more likely to agree with these items. Items 4–6 (ethical respect) show that even if individuals have higher ethical safety scores are still unlikely to agree with these statements. This indicates that items are differentiating between individual attitudes towards ethical safety, which is in accordance with previous research [4].

Most participants showed the highest agreement on items 1 and 2. However, there are no trivial statements which received ‘strongly agree’ or ‘strongly disagree’ scores from all participants. These findings indicate that all items are relevant to the overall evaluation of ethical safety. The DIF results show no notable differences between groups, indicating that the ESQ items function similarly across the different demographic groups, supporting the ESQ’s validity across diverse populations of healthcare professionals and demographic variables. However, this needs to be interpreted with caution given the subgroups are heavily skewed towards females (*n* = 234), nurses (*n* = 221) and bachelor’s degree-qualified professionals (*n* = 125). This can lead to differences in response patterns that are also attributed to the cultural context of the items and not the actual construct under evaluation.

### Limitations

Selection of participants using purposive sampling can be a limitation of this validation process, however specific target populations were required in the study [29]. Although the population included is adequate for analysis, there are several attributes that are distinct for populations of nurses and physiotherapists in different countries and, therefore, the transferring or extrapolation of the results and their interpretation to other contexts or populations require caution. Although the sample was representative of the population of nurses and physiotherapists in the European Union, there was a much higher representation of nurses compared to physiotherapists and predominantly female participants. Both disbalances are, however, consistent with the general populations that the samples represent. The sample size whilst small, is still within an adequate sample size indicated for studies of this nature [12]. The small sample size limited the possibility to perform a robust confirmatory factor analysis which is recommended in future research with a larger sample size.

## Conclusion

The results of this validation study suggest that the ESQ is a psychometrically sound, reliable and valid instrument to measure ethical safety in nurses and physiotherapists in acute healthcare environments. The general ethical safety statements within the ESQ can, according to the analysis carried out, be removed from the tool without influencing the validity of the overall scale. Ethical safety is considered to be a necessary feature of acute healthcare environments, which allows healthcare professionals the space to verbalise ethical issues in practice and apply the necessary ethical decisions within such environments. The ESQ can be used in future research to quantify the perceptions of healthcare professionals regarding their healthcare environments and to what extent these are safe spaces for ethical deliberation. Studies which seek to generate evidence that may assess educational environments and contribute towards the development of policies that support increasingly safe, practice and educational settings may also employ the use of the ESQ in the future. Thus, overall, the ESQ offers valuable insights about ethical safety research in healthcare.

## Data Availability

The datasets used and/or analysed during the current study are available from the corresponding author on reasonable request.
